# Supplementary material for: Combining Celiac and Hepatic Vagus Nerve Neuromodulation Reverses Glucose Intolerance and Improves Glycemic Control in Pre- and Overt-Type 2 Diabetes Mellitus
Source: Biomedicines. 2023 Sep 4;11(9):2452. doi: 10.3390/biomedicines11092452 (PMC10525327; doi:10.3390/biomedicines11092452)
Supplement: Supplementary file 1 [file biomedicines-11-02452-s001.zip › biomedicines-2559858-supplementary.pdf]

Supplementary Section

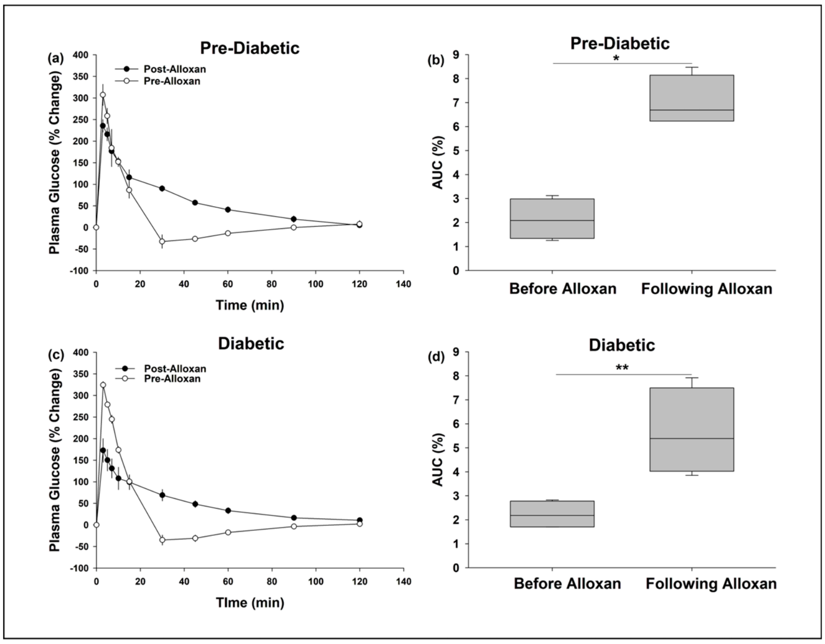

Figure S1: Injection of alloxan significantly decreased glycemic control as assessment with percent change in PG in swine that were characterized as pre-diabetic and diabetic. Due to possible confounding effects of an increase in baseline following alloxan, PG was normalized to baseline by calculating the percent change. Of interest, the percent change of the peak glucose response during both pre-diabetic and diabetic IVGTTs was lower following alloxan in both pre-diabetic and diabetic groups (a) and (c). This was due to an increased baseline following alloxan in both groups with corresponding similar peak responses. Slower recovery rates can be observed following alloxan in both groups, indicative of decreased glucose tolerance. AUC of the percent change demonstrated glucose intolerance following alloxan in pre-diabetic (b) and diabetic (d) swine. * p=0.03 and ** p=0.02, Mann–Whitney U test.

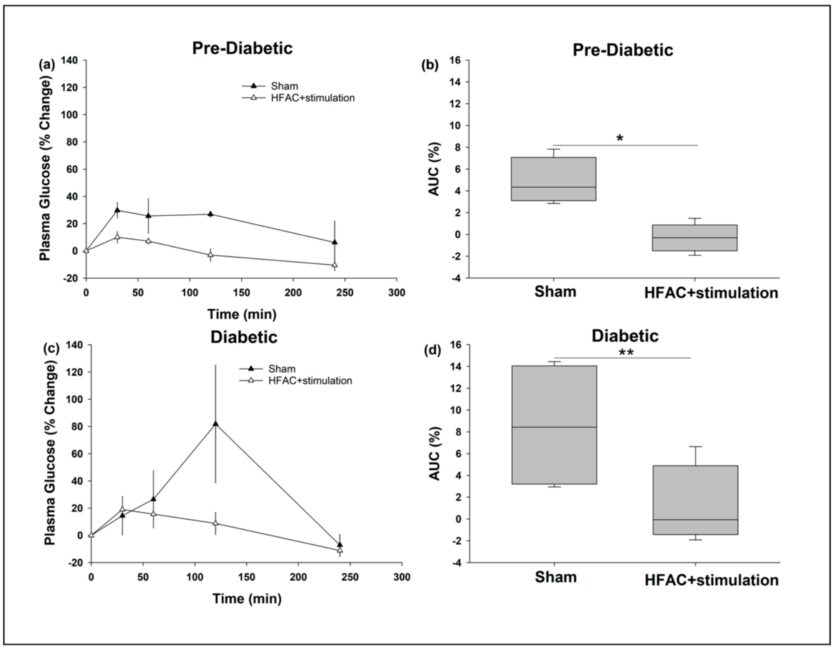

Figure S2: A significant increase in glycemic control was observed during OGTTs by assessment of percent change in PG in swine that were characterized as pre-diabetic and diabetic. Due to possible confounding effects of a decreased in baseline during OGTTs, PG was normalized to baseline by calculating the percent change. (a) Following ingestion of glucose in pre-diabetic swine in Sham conditions, PG increased approximately 30% by 30 min which remained elevated during 2 hours of the OGTT. The application of HFAC+stimulation during the OGTT in pre-diabetic swine significantly blunted this increase (b). (c) Following ingestion of glucose in diabetic swine in Sham conditions, PG increased and peaked at an increase of approximately 80%. The application of HFAC+stimulation during the OGTT in diabetic swine significantly blunted this increase (d). *p=0.004 and ** p<0.05, Mann–Whitney U test.
